# CXC chemokine ligand 12/Stromal cell-derived factor-1 regulates cell adhesion in human colon cancer cells by induction of intercellular adhesion molecule-1

**DOI:** 10.1186/1423-0127-19-91

**Published:** 2012-10-25

**Authors:** Shui-Yi Tung, Shun-Fu Chang, Ming-Hui Chou, Wen-Shih Huang, Yung-Yu Hsieh, Chien-Heng Shen, Hsing-Chun Kuo, Cheng-Nan Chen

**Affiliations:** 1 Department of Hepato-Gastroenterology, Chang Gung Memorial Hospital, Chiayi, Taiwan; 2 Biophotonics & Molecular Imaging Research Center, National Yang Ming University, Taipei, Taiwan; 3 Department of Biochemical Science and Technology, National Chiayi University, Chiayi, Taiwan; 4 Division of Colon and Rectal Surgery, Department of Surgery, Chang Gung Memorial Hospital, Chiayi, Taiwan; 5 Department of Nursing, Chang Gung University of Science and Technology; Chronic Diseases and Health Promotion Research Center, CGUST, Chiayi, Taiwan

**Keywords:** Colorectal cancer, Stromal cell-derived factor-1, Intercellular adhesion molecule-1, Cell adhesion, Transcriptional regulation

## Abstract

**Background:**

The CXC chemokine ligand 12 (CXCL12)/stromal cell-derived factor-1 (SDF-1) and CXC receptor 4 (CXCR4) axis is involved in human colorectal cancer (CRC) carcinogenesis and can promote the progression of CRC. Interaction between CRC cells and endothelium is a key event in tumor progression. The aim of this study was to investigate the effect of SDF-1 on the adhesion of CRC cells.

**Methods:**

Human CRC DLD-1 cells were used to study the effect of SDF-1 on intercellular adhesion molecule-1 (ICAM-1) expression and cell adhesion to endothelium.

**Results:**

SDF-1 treatment induced adhesion of DLD-1 cells to the endothelium and increased the expression level of the ICAM-1. Inhibition of ICAM-1 by small interfering RNA (siRNA) and neutralizing antibody inhibited SDF-1-induced cell adhesion. By using specific inhibitors and short hairpin RNA (shRNA), we demonstrated that the activation of ERK, JNK and p38 pathways is critical for SDF-1-induced ICAM-1 expression and cell adhesion. Promoter activity and transcription factor ELISA assays showed that SDF-1 increased Sp1-, C/EBP-β- and NF-κB-DNA binding activities in DLD-1 cells. Inhibition of Sp1, C/EBP-β and NF-κB activations by specific siRNA blocked the SDF-1-induced ICAM-1 promoter activity and expression. The effect of SDF-1 on cell adhesion was mediated by the CXCR4.

**Conclusion:**

Our findings support the hypothesis that ICAM-1 up-regulation stimulated by SDF-1 may play an active role in CRC cell adhesion.

## Background

Colorectal cancer (CRC) is one of the most common malignancies and is the fourth leading cause of cancer death worldwide. Death due to colorectal cancer usually results from metastatic spread of cancer cells [1]. Chemokines play a pivotal role in tumor progression because they may affect growth, adhesion and metastasis of tumor cells [2,3]. CXC chemokine ligand 12 (CXCL12)/Stromal cell-derived factor (SDF-1) is a member of the CXC chemokine family, which plays an important role in chemotaxis, haematopoiesis, angiogenesis and tumor spread and metastasis [4,5]. SDF-1 is expressed in several cancer cells and is involved in tumor cell migration and metastasis [6,7]. Several studies have indicated that CRC cells promoted their survival and migration to distant tissues by interaction between SDF-1 and its specific receptor, CXC receptor 4 (CXCR4). The higher expression of CXCR4 in tumor tissue correlates with poor prognosis and poor survival in CRC patients [8,9].

Tumor cell adhesion is not only associated in tumor cell detachment from the primary carcinoma, but is also involved in cell attachment to distant tissue [10]. The maintenance and promotion of cell adhesion are particularly important processes in CRC progression and metastasis. The intercellular adhesion molecule-1 (ICAM-1) is a member of the immunoglobulin (Ig) gene superfamily of adhesion molecules. It is involved in cancer cell adhesion and in the immune responses of tumors [11,12]. The interaction between ICAM-1 and its specific ligand may facilitate adhesion of cancer cells to the vascular endothelium and subsequently in the promotion of metastasis. It has been reported that the serum level of soluble (s)ICAM-1 is elevated in patients with cancers [13]. High serum levels of sICAM-1 have been shown to be correlated with tumor progression in cases of CRC and associated with liver metastasis [14,15]. Normal colon tissues do not express ICAM-1, but this adhesion molecule can be up-regulated on malignant transformation [16]. Several studies also suggest that ICAM-1 plays an important role in CRC progression and metastasis [17-19]. It has been demonstrated that SDF-1 effectively induced the expression of urokinase plasminogen activator in CRC cells [20]. In addition, SDF-1 has been implicated in integrin-mediated adhesion in lung cancer and renal cancer cells [21-23]. SDF-1–mediated cell adhesion via ICAM-1 has also been reported in leukemia cells [24]. Firm cell adhesion between CD34^+^ cells and endothelial cells is reported to occur when ICAM-1 and SDF-1 are co-immobilized [25]. However, it is not clear whether the regulation of ICAM-1 expression and cell adhesion are mediated by SDF-1 in colorectal cancer cells.

Mitogen-activated protein kinases (MAPKs) are known as major signaling pathways that play an essential role in the development of inflammation and cancer [26]. Furthermore, they have been reported to be involved in cancer cell pathological functions, including invasion and angiogenesis [27]. In the present study, we show that SDF-1 induces ICAM-1 expression in colorectal cancer DLD-1 cells. Our data indicate that the SDF-1-induced increase of ICAM-1 expression and cell adhesion is mediated through the ERK, JNK and p38 intracellular signaling cascades, and the transcription factors Sp1, C/EBP-β and NF-κB. Our findings provide evidence of the molecular mechanisms by which SDF-1 induces ICAM-1 expression in colorectal cancer cells.

## Methods

### Materials

All culture materials were purchased from Gibco (Grand Island, NY, USA). PD98059 (ERK inhibitor), SP600125 (JNK inhibitor), and SB203580 (p38 inhibitor) were purchased from Calbiochem (La Jolla, CA). Polyclonal anti-ICAM-1 and anti-CXCR4 neutralizing antibody were obtained from R & D Systems (Minneapolis, MN). Mouse monoclonal antibodies (mABs) against ERK2, JNK1, phospho-ERK1/2, phospho-JNK1/2, NF-κB p65, Sp1, and C/EBP-β were purchased from Santa Cruz Biotechnology (Santa Cruz, CA). Rabbit polyclonal antibodies against p38 and mouse monoclonal phospho-p38 antibody were purchased from Cell Signaling Technology (Beverly, MA). ERK, JNK, and p38 shRNA and control shRNA (scrambled negative control containing random DNA sequences) were purchased from the National RNAi Core Facility in Academic Sinica, Taipei, Taiwan. The NF-κB p65, Sp1, and C/EBP-β-siRNA and control siRNA (scrambled negative control containing random DNA sequences) were purchased from Invitrogen (Carlsbad, CA). AMD3100 (CXCR4 inhibitor) and all other chemicals of a reagent grade were obtained from Sigma (St. Louis, MO).

### Cell culture

The colon cancer DLD-1 and SW48 cell lines were purchased from the Bioresources Collection and Research Center (BCRC) of the Food Industry Research and Development Institute (Hsinchu, Taiwan). Cells were maintained in Dulbecco’s Modified Eagle Medium (DMEM) supplemented with 10% fetal bovine serum (FBS) and 1% penicillin/streptomycin in a CO_2_ incubator at 37°C.

Human umbilical vein endothelial cells (HUVECs) were isolated by collagenase digestion. Briefly, human umbilical cords were washed, filled with 0.1% collagenase, and incubated at 37°C for 10 min. The suspension of ECs collected was centrifuged for 10 min at 1200 × g. The pellet was resuspended in M199 supplemented with penicillin/streptomycin and 20% FBS, after which the cells were plated onto cell culture dishes. Cultures were maintained at 37°C in a humidified atmosphere containing 5% CO_2_. HUVECs of passage 2 were used for experiments.

### Flow cytometry analysis

The expressions of CXCR4 and ICAM-1 on the surface of DLD-1 cells were measured by indirect immunofluorescence using flow cytometry and the mouse monoclonal antibody (20 μg/mL; R&D) against CXCR4 and ICAM-1 [28].

### Cell adhesion assay

Adherence of DLD-1 and SW48 cells to HUVECs was determined as described previously [29]. Before the adhesion experiments, cells were cultured in low-serum medium (0.5% FBS) for 12 h and then treated with SDF-1 (10 ng/mL) for 4 h and labeled with 1, 10-dioctadecyl-3, 3, 30, 30-tetramethylindocarbocyanine (DiI; Molecular Probes, Eugene, OR) for 20 min. The labeled cells (2 × 10^5^ cells/mL) were added to HUVECs and incubated for 1 h. In parallel experiments, DLD-1 cells were treated with specific inhibitors or transfected with specific shRNAs during SDF-1 stimulation. Non-adherent cells were removed by washing with PBS. The adherent cancer cells on the EC surface were identified and counted in 10 randomly selected microscopic fields (1.37 mm × 1.07 mm) under a Nikon Ti-E inverted epifluorescence microscope with ×10 objective, and the adhesion was expressed as a multiple compared to the controls. To determine the role of CXCR4 and ICAM-1 in the adhesion of DLD-1 and SW48 cells to HUVECs, CRC cells were pre-incubated with 20 μg/mL of anti-human CXCR4 and ICAM-1 antibodies (R&D) for 30 min, pretreated with AMD3100, or transfected with the ICAM-1 siRNA prior to the adhesion assay.

### Real-time quantitative PCR

Real-time PCR of three transcripts was performed using an ABI Prism 7900HT with the FastStart DNA SYBR Green I kit (Roche). The designed primers in this study were: ICAM-1 forward primer, 5^′^- GTGAC ATGCA GCACC TCCTG-3^′^; ICAM-1 reverse primer, 5^′^- TCCAT GGTGA TCTCT CCTCA-3^′^; 18S rRNA forward primer, 5^′^-CGGCG ACGAC CCATT CGAAC-3^′^, 18S rRNA reverse primer, 5^′^-GAATC GAACC CTGAT TCCCC GTC-3^′^. Quantification was performed using the 2^−ΔΔCt^ method [30]. All samples were measured in duplicate. The average value of both duplicates was used as the quantitative value.

### ELISA for cell surface ICAM-1 expression

ICAM-1 expression on the cancer cell surface was measured by cell surface ELISA as previously described [29]. Briefly, DLD-1 cells cultured in 96-well plates were fixed by 4% paraformaldehyde. Cell surface ICAM-1 expression was assessed using the mouse anti-human ICAM-1 mAb, followed by a horseradish-peroxidase-conjugated secondary antibody. The absorbance of each well was measured at 490 nm after the reactions were stopped.

### Western blot analysis

Samples were lysed with a buffer containing 1% NP-40, 0.5% sodium deoxycholate, 0.1% SDS, and a protease inhibitor mixture (PMSF, aprotinin and sodium orthovanadate). Protein concentration was determined using the Bio-Rad protein assay kit (Bio-Rad, Hercules, CA). Equal amounts of total proteins were separated by SDS-polyacrylamide gel electrophoresis (PAGE) (10% running, 4% stacking), transferred onto a nitrocellulose membrane, and analyzed using the designated antibodies and the Western-Light chemiluminescent detection system (Bio-Rad).

### shRNA and siRNA transfection

For shRNA transfection, DLD-1 cells were transfected with the designated MAPK shRNA plasmids by using shRNA plasmid transfection reagent (Santa Cruz). For siRNA transfection, DLD-1 cells were transfected with the specific NF-κB p65, Sp1-1, and C/EBP-β-siRNA or control siRNA by using an RNAiMAX transfection kit (Invitrogen).

### Luciferase assays

Human ICAM-1 promoter constructs containing −537/+16, -413/+16, -323/+16, -245/+16, -134/+16, and −88/+16 of ICAM-1 5'-flanking DNA linked to the firefly luciferase reporter gene of plasmid pGL4 (Promega, Madison, WI) were used. DNA plasmids at a concentration of 1 mg/ml were transfected into DLD-1 cells by Lipofectamine (Gibco). The pSV-β-galactosidase plasmid was cotransfected to normalize the transfection efficiency. Values obtained were normalized to the levels of β-galactosidase in the cell lysates. β-galactosidase activities were determined with an assay kit and exhibited <20% variation between samples.

### Transcription factor assays (TF ELISA assays)

Nuclear extracts of cells were prepared by nuclear protein extract kits (Panomics, Redwood City, CA). Equal amounts of nuclear proteins were used for quantitative measurements of NF-κB p65, Sp1 and C/EPB-β activation using commercially available ELISA kits (Panomics).

### Chromatin immunoprecipitation assay (ChIP)

After cross-linking with 1% formaldehyde, the cells were centrifuged and then resuspended in a lysis buffer for three times of sonication of 15 sec each. Supernatants were recovered by centrifugation. Aliquots of the precleared sheared chromatin were then immunoprecipitated using 2 μg antibodies against IgG, NF-κB p65, Sp1 and C/EPB-β. The resulting DNA was used for PCR analysis, and the amplified DNA fragments were visualized on an agarose gel. The human ICAM-1 promoter region −305/-91 was amplified with the PCR primer pairs 5^′^-ACCTT AGCGC GGTGT AGACC-3^′^ and 5^′^-CTCCG GAACA AATGC TGC-3^′^.

### Statistical analysis

The results are expressed as the mean ± standard error of the mean (SEM). Statistical analysis was determined using an independent Student t-test for two groups of data and analysis of variance (ANOVA) followed by Scheffe’s test for multiple comparisons. *P* values less than 0.05 were considered significant.

## Results

### Effect of SDF-1 on adhesion of CRC cells to HUVECs

**Figure 1:**
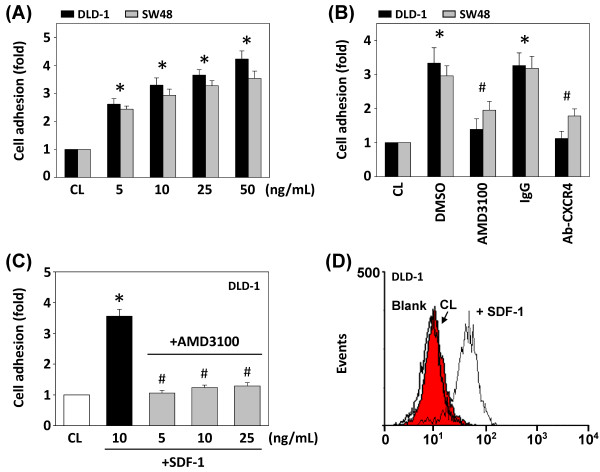
**Effect of SDF-1 on CRC cell adhesion to HUVECs.** All bar graphs represent the multiple increases over the control cells (CL), calculated as the mean ± standard error of the mean (SEM) from five experiments. DLD-1 cells were kept as CL or stimulated with SDF-1 for 4 h. *P < 0.05 versus CL. ^#^*P* < 0.05 versus vehicle control (DMSO) or IgG-treated cells with SDF-1 stimulation. (**A**) DLD-1 and SW48 cells treated with different doses of SDF-1 were labeled with DiI and added to confluent monolayers of HUVECs for 1 h. (**B**) Before being kept as CL or stimulated with SDF-1, DLD-1 and SW48 cells were pretreated with AMD3100, or incubated with neutralizing antibody against CXCR4 for 1 h. (**C**) Before being kept as CL, DLD-1 cells were pretreated with AMD3100 for 1 h and were then stimulated with different doses of SDF-1. (**D**) Flow cytometry shows the cell surface expression of CXCR4. DLD-1 cells were kept as CL or stimulated with SDF-1. Cells were incubated with mAb against CXCR4 or isotype-matched IgG served as a blank.

In order to quantify the adhesion of the CRC cells to HUVECs, DLD-1 and SW48 cells were treated with different doses of SDF-1 (0–50 ng/mL) for 4 h and then labeled with DiI. The labeled cells were seeded onto the HUVEC monolayers and co-cultured for 1 h. After removal of the non-adherent cells, the remaining adherent cells were evaluated. SDF-1 stimulation induced increased adherence of DLD-1 and SW48 cells to HUVECs in a dose-dependent manner (Figure 1A). To assess the role of CXCR4 in SDF-1-induced cell adhesion in DLD-1 and SW48 cells, we evaluated the effect of CXCR4 inhibitor AMD3100 (100 nM) and CXCR4 neutralizing antibody on SDF-1-induced cell adhesion. Pretreatment of AMD3100 and neutralizing antibody against CXCR4 significantly inhibited the adhesion of DLD-1 and SW48 cells to HUVECs (Figure 1B).

Pretreatment with AMD3100 could also inhibit adhesion of DLD-1 cells treated with different doses of SDF-1 (Figure 1C). The effect of SDF-1 on CXCR4 expression in DLD-1 cells was evaluated by flow cytometry assay using CXCR4 antibody. As shown in Figure 1D, CXCR4 is hardly expressed in DLD-1 cells, but cell surface expression is up-regulated by stimulation with SDF-1 for 4 h in DLD-1 cells.

### Effect of SDF-1-induced ICAM-1 expression on adhesion of DLD-1 cells to HUVECs

**Figure 2:**
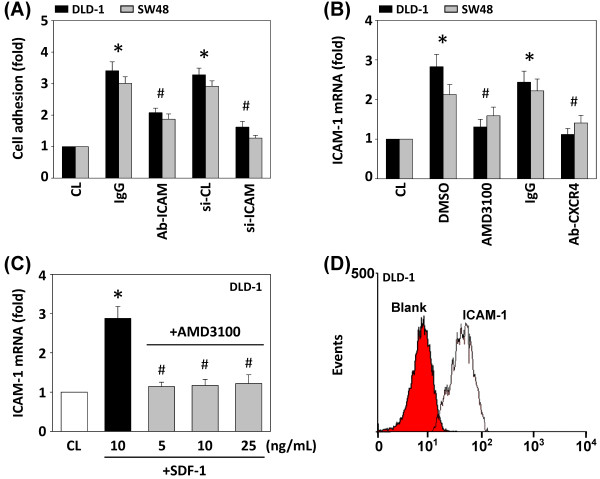
**Effect of SDF-1 on ICAM-1 expression and cell adhesion of CRC cells.** (**A**) SDF-1-stimulated DLD-1 and SW48 cells were treated with isotype-matched IgG or 20 μg/mL neutralizing antibody against ICAM-1, or were transfected with control siRNA (si-CL) or si-ICAM-1, and were then added to HUVECs for 1 h. (**B**) DLD-1 and SW48 cells were kept as CL or stimulated with SDF-1 for 4 h. Before being kept as CL or stimulated with SDF-1, cells were pretreated with AMD3100 or incubated with neutralizing antibody against CXCR4 for 1 h. (**C**) Before being kept as CL, DLD-1 cells were pretreated with AMD3100 for 1 h, and were then stimulated with different doses of SDF-1 for 4 h. All bar graphs represent multiple increases of CL cells normalized to 18S rRNA. *p < 0.05 versus CL. ^#^*p* < 0.05 versus IgG-treated, DMSO-treated, or si-CL-transfected cells. (**D**) The ICAM-1 basal expression level of DLD-1 cells was analyzed by flow cytometry. Cells were incubated with mAb against ICAM-1 or isotype-matched IgG served as a blank.

To investigate the role of ICAM-1 in the adhesion of DLD-1 and SW48 cells to HUVECs, we blocked the ICAM-1 function by using ICAM-1 neutralizing antibody and specific siRNA. SDF-1-induced adhesion of DLD-1 and SW48 cells to HUVECs was significantly inhibited by cells incubated with ICAM-1 neutralizing antibody, or transfected with ICAM-1 siRNA, suggesting a direct involvement of ICAM-1 in the adhesive interaction between CRC cells and HUVECs (Figure 2A). To assess the role of CXCR4 in SDF-1-induced ICAM-1 expression in DLD-1 and SW48 cells, we evaluated the effect of AMD3100 (100 nM) and CXCR4 neutralizing antibody on SDF-1-induced ICAM-1 expression. Pretreatment of AMD3100 and neutralizing antibody against CXCR4 markedly inhibited the expression of ICAM-1 mRNA (Figure 2B).

Pretreatment with AMD3100 could also inhibit ICAM-1 expression of DLD-1 cells treated with different doses of SDF-1 (Figure 2C). The basal level of ICAM-1 expression in DLD-1 cells was evaluated by flow cytometry assay, as shown in Figure 2D.

### SDF-1-induced ICAM-1 expression in DLD-1 and SW48 cells is dose- and time-dependent

**Figure 3:**
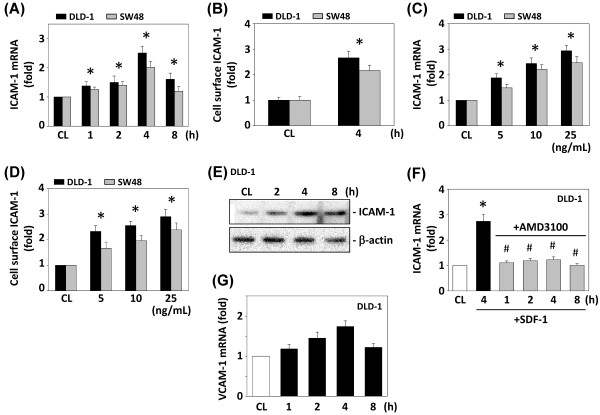
**Induction of ICAM-1 expression in DLD-1 and SW48 cells by SDF-1 stimulation.** Samples were isolated at the indicated time periods or doses. All bar graphs represent multiple increases of control cells (CL) normalized to 18S rRNA by real-time PCR analysis (**A, C**). The cell surface ICAM-1 protein expression levels were determined by cell surface ELISA analyses (**B, D**). DLD-1 and SW48 cells were stimulated with 10 ng/mL SDF-1 for the times indicated (**A, B**), or stimulated with SDF-1 at various doses for 4 h (**C, D**). Data are shown as mean ± SEM. *P < 0.05 versus CL DLD-1 cells. (**E**) The expression of total ICAM-1 in DLD-1 cell lysate after SDF-1 stimulation for the times indicated was determined using Western blotting. (**F**) Before being kept as CL, cells were pretreated with AMD3100 for 1 h and were then stimulated with SDF-1 for the times indicated. *p < 0.05 versus CL. ^#^*p* < 0.05 versus DMSO-treated cells. (**G**) DLD-1 cells were stimulated with SDF-1 for the times indicated. The VCAM-1 mRNA expression level was determined by real-time PCR analysis normalized to 18S rRNA.

Next, we examined the effect of SDF-1 on the ICAM-1 mRNA and cell surface protein expression by CRC DLD-1 and SW48 cells. Cells were stimulated with SDF-1 (10 ng/mL) for the times indicated, or at different doses (0–50 ng/mL) for 4 h. The changes in ICAM-1 expression compared with the control cells at the same time points were analyzed by real-time PCR, and cell surface ICAM-1 expression was detected by ELISA. The ICAM-1 mRNA level began to increase after 1 h of SDF-1 treatment and reached its highest level at 4 h (Figure 3A). The cell surface ICAM-1 expression of DLD-1 and SW48 cells also increased after 4 h of SDF-1 stimulation (Figure 3B). The induction of ICAM-1 mRNA and cell surface protein expression by SDF-1 stimulation was dose-dependent (Figure 3C and 3D). In addition, SDF-1 also induced an increase in total ICAM-1 protein expression in DLD-1 cells in a time-dependent manner (Figure 3E).

ICAM-1 expression in the indicated times was inhibited by DLD-1 cells treated with AMD3100 (Figure 3F). In addition, SDF-1 induced only slight increases in the DLD-1 VCAM-1 transcript level compared with untreated cells (Figure 3G).

### Effect of MAPK inhibitors on ICAM-1 mRNA expression and cell adhesion in DLD-1 cells

**Figure 4:**
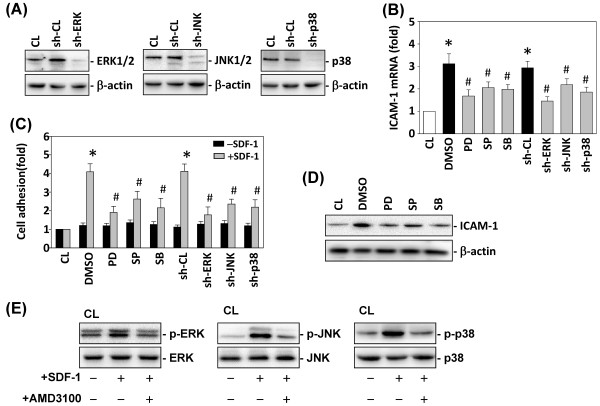
**MAPK pathways are required for SDF-1-induced ICAM-1 expression and cell adhesion.** DLD-1 cells were kept as CL or stimulated with 10 ng/mL SDF-1 for 4 h. Before being kept as CL or stimulated with SDF-1, DLD-1 cells were pretreated with PD98059 (PD), SP600125 (SP) or SB203580 (SB) individually for 1 h, or transfected with control shRNA (sh-CL), or a specific shRNA of sh-ERK, sh-JNK or sh-p38. (**A**) The gene silencing efficiency of 48-h transfection of shRNA on ERK, JNK, and p38 protein levels of DLD-1 cells. After 48 h of transfection, protein was isolated and the ERK, JNK, and p38 expression was analyzed by Western blotting. (**B**) All bar graphs represent multiple increases of CL DLD-1 cells normalized to 18S rRNA. (**C**) DLD-1 cells were stimulated with or without SDF-1 for 4 h, and were added to HUVECs for 1 h. The results are shown as mean ± SEM. *P < 0.05 versus CL. ^#^*P* < 0.05 versus vehicle control (DMSO) or control shRNA (sh-CL) with SDF-1 stimulation. (**D**) DLD-1 cells were kept as CL, pretreated with DMSO, PD98059 (PD), SP600125 (SP), or SB203580 (SB), and stimulated with SDF-1 for 4 h. The total ICAM-1 protein expressions were determined by Western blot analysis. (**E**) DLD-1 cells were kept as CL, pretreated with AMD3100, and stimulated with SDF-1; the phosphorylation of ERK, JNK and p38 was determined by Western blotting.

To determine whether the SDF-1-induced ICAM-1 expression and cell adhesion to HUVECs is mediated through the MAPK-dependent pathway, DLD-1 cells were incubated with the specific inhibitor for ERK (PD98059; 30 μM), JNK (SP600125; 20 μM), or p38 (SB203580; 10 μM) for 1 h before and during stimulation with SDF-1. The SDF-1-1-induced ICAM-1 mRNA expression in DLD-1 cells (Figure 4B) and cell adhesion to HUVECs (Figure 4C) was significantly inhibited by PD98059, SP600125 and SB203580. To further confirm the involvement of ERK, JNK, and p38, in the modulation of ICAM-1 expression through SDF-1 stimulation, we examined the effects of the specific shRNAs of these signaling pathways on SDF-1-induced ICAM-1 expression in DLD-1 cells. The effectiveness of the silencing was validated because ERK-, JNK-, and p38-shRNAs (compared with control shRNA) caused an 85% reduction in ERK, JNK, and p38 protein expressions, respectively (Figure 4A). The SDF-1-induced ICAM-1 mRNA (Figure 4B) and cell surface ICAM-1 (Figure 4C) were inhibited by transfection with ERK-, JNK-, and p38-shRNAs. Furthermore, SDF-1-induced total ICAM-1 protein expression in DLD-1 cells was also inhibited by cells pretreated with PD98059, SP600125 and SB203580 (Figure 4D).

Our previous study demonstrated that the phosphorylation level of p38 in DLD-1 cells increased rapidly after stimulation by SDF-1 [20]. In the present study, the phosphorylation of ERK, JNK and p38 in DLD-1 cells also increased after SDF-1 stimulation (Figure 4E). In addition, the SDF-1–induced phosphorylation of ERK, JNK and p38 in DLD-1 cells was inhibited by cells pretreated with AMD3100 (Figure 4E).

### Sp1, C/EBP and NF-κB are essential for the SDF-1-induction of human ICAM-1 expression

**Figure 5:**
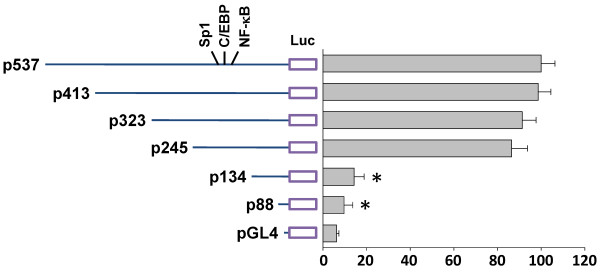
**The roles of Sp1, C/EBP-β and NF-κB in SDF-1-induced ICAM-1 mRNA expression and promoter activity.** The ICAM-1 promoter p537-Luc plasmid and deletion promoter constructs. DLD-1 cells were cotransfected with 5^′^-deletion constructs and stimulated with 10 ng/mL SDF-1 for 4 h. ICAM-1 promoter activity was measured using a luciferase assay normalized to β-galactosidase activity and was shown to be relative to that of DLD-1 cells transfected with p537-Luc (set to 100%). *P < 0.05 versus p537-Luc.

To identify the *cis*-acting elements in the ICAM-1 gene promoter that mediate SDF-1-induced ICAM-1 transcription, luciferase assays were conducted with the ICAM-1 p537-Luc plasmid and several deletion promoter constructs (Figure 5). In DLD-1 cells, the −537/+16 region of ICAM-1 directed maximum luciferase activity. Sequence deletion from −245 to −134 caused the luciferase activity to decrease to ~85%; and 5'-deletion to −88 nearly eliminated the activity (Figure 5).

**Figure 6:**
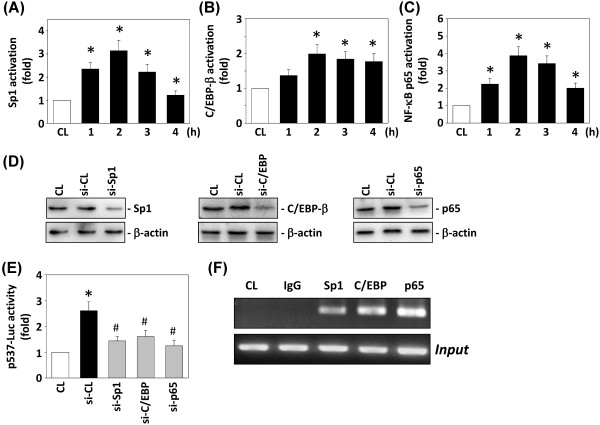
**Sp1-, C/EBP-β- and NF-κB-DNA binding activities induced by SDF-1 stimulation in DLD-1 cells.** (**A**) Sp1 activation, (**B**) C/EBP-β activation and (**C**) NF-κB p65 activation were determined by a TF ELISA assay. (**D**) The gene silencing efficiency of 48-h transfection of siRNA on Sp1, C/EBP-β, and p65 protein levels of DLD-1 cells. After 48 h of transfection, protein was isolated and the Sp1, C/EBP-β, and p65 expression was analyzed by Western blotting. (**E**) ICAM-1 mRNA and p537-Luc activity were determined in DLD-1 cells transfected with control siRNA (si-CL), si-Sp1, si-C/EBP-β, or si-p65, and then stimulated with 10 ng/mL SDF-1 for 4 h. All bar graphs represent folds of CL DLD-1 cells, mean ± SEM. *P < 0.05 versus CL. ^#^*P* < 0.05 versus si-CL with SDF-1 stimulation. (**F**) ChIP assays were performed for Sp1, C/EBP-β, and NF-κB using p65 antibodies.

To investigate whether SDF-1 can activate Sp1, C/EBP-β, and NF-κB in DLD-1 cells, we performed quantitative analysis of the Sp1, C/EBP-β, and NF-κB p65 binding activities *in vitro* using TF ELISA kits from Panomics. The treatment of DLD-1 cells with SDF-1 caused Sp-1- (Figure 6A), C/EBP-β- (Figure 6B) and NF-κB p65-DNA (Figure 6C) binding activities to increase after 1 h and remain elevated for at least 2 hours.

We further tested whether Sp1, C/EBP-β, and NF-κB activation are involved in the signal transduction pathway leading to the SDF-1 induction of ICAM-1promoter activity. DLD-1 cells were transfected with siRNAs for Sp1, C/EBP-β, or p65 followed by stimulation with SDF-1 for 4 h. The Sp1, C/EBP-β, and NF-κB p65-specific siRNAs (compared with control siRNA) caused an 80% reduction in Sp1, C/EBP-β, and NF-κB p65 protein expressions, respectively (Figure 6D). The SDF-1-induced ICAM-1 p537-Luc promoter activity levels (Figure 6E) were significantly down-regulated by the inhibition of specific siRNAs, indicating that Sp1, C/EBP-β, and p65 are involved in the regulation of ICAM-1 gene expression. These results were confirmed by ChIP analysis. Immunoprecipitated chromosomal DNA with Sp1, C/EBP-β and p65 antibodies was subjected to PCR, using primers designed to amplify the ICAM-1 promoter region harboring these transcription factor binding sites. Sp1, C/EBP-β and NF-κB were indeed found to bind the ICAM-1 promoter region (Figure 6F).

### MAPK signaling pathways were involved in SDF-1-induced ICAM-1 promoter activity

**Figure 7:**
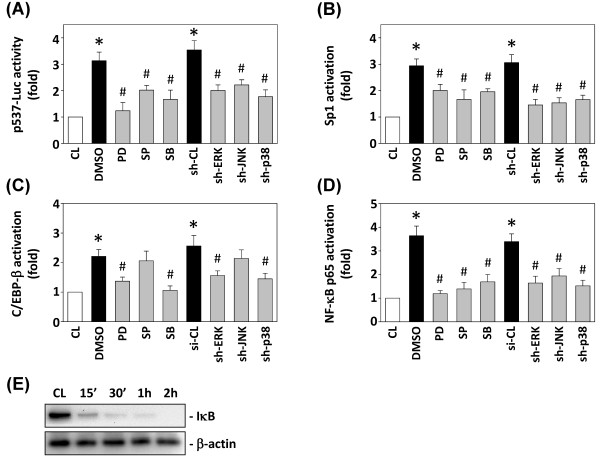
**MAPK signaling pathways are involved in SDF-1-induced ICAM-1 promoter activity.** (**A**) ICAM-1 p537-Luc activity was determined in DLD-1 cells pretreated with vehicle (DMSO), or PD98059 (PD), SP600125 (SP), and SB203580 (SB) individually for 1 h, or transfected with control shRNA (sh-CL), or a specific shRNAs of ERK (sh-ERK), JNK (sh-JNK) or p38 (sh-p38), and then stimulated with 10 ng/mL SDF-1 for 4 h. (**B**) Sp1, (**C**) C/EBP-β and (**D**) NF-κB p65 activation were determined by TF ELISA assays in DLD-1 cells pretreated with vehicle (DMSO), or PD98059 (PD), SP600125 (SP) and SB203580 (SB) individually for 1 h, or transfected with sh-CL, or sh-ERK, sh-JNK or sh-p38, and were then stimulated with SDF-1 for 2 h. All bar graphs represent multiple increases of CL DLD-1 cells, mean ± SEM. *P < 0.05 versus CL. ^#^*P* < 0.05 versus vehicle control (DMSO) with SDF-1 stimulation. (**E**) DLD-1 cells were kept as CL or stimulated with SDF-1 for the times indicated, and the IκB protein expression was determined by Western blot analysis.

To evaluate whether the inhibition of ICAM-1 expression by MAPK signaling pathways occurs at the transcriptional level, we studied the effects of MAPK inhibitors and shRNAs on SDF-1-induced ICAM-1 p537-Luc promoter activity. Treatment of SDF-1 increased the luciferase activity compared with the unstimulated cells after normalization through transfection control (Figure 7A). Pretreatment of cells with PD98059, SP600125 and SB203580, or ERK-, JNK-, p38-shRNA resulted in a marked inhibition of the SDF-1-induced ICAM-1 promoter activity (Figure 7A).

To explore whether MAPKs activate the promoter leading to ICAM-1 transcription via activations of Sp1, C/EBP-β, and NF-κB, DLD-1 cells were pretreated with MAPK inhibitors or transfected with MAPK shRNAs followed by the SDF-1 treatment. The Sp1, C/EBP-β, and NF-κB p65 activations were assessed using TF ELISA kits. Pretreatment of cells with PD98059, SP600125 and SB203580, or transfected with ERK-, JNK-, and p38-shRNA significantly inhibited the SDF-1-induced Sp1- (Figure 7B) and NF-κB p65-DNA (Figure 7D) binding activity, whereas pretreatment of cells with PD98059 and SB203580, or transfected with ERK- and p38-shRNA markedly inhibited the SDF-1-induced C/EBP-β-DNA binding activity (Figure 7C). In addition, as shown in Figure 7E, SDF-1 also induced degradation of IκB protein in DLD-1 cells in a time-dependent manner.

## Discussion

The cell adhesion process plays a key role in tumor progression. Several adhesion molecules, including ICAM-1, have been implicated in the tumor transformation and metastasis via the mediating adhesion of cancer cells to the vascular endothelium. This is an important procedure for executing the subsequent steps of invasion and survival of cancer cells [31]. It has been shown that CRC metastases are related to the ability of cancer cells to adhere to the microvascular endothelium of the lung that expresses the ligands for ICAM-1 on the tumor cell surface [32]. Previous studies demonstrated that ICAM-1 is expressed in CRC cells, and patients with higher ICAM-1 levels in plasma reveal poor prognosis compared to healthy controls [14,17,33]. In addition, up-regulation of ICAM-1 expression is correlated with the cancer stage and distant metastasis, suggesting that ICAM-1 plays a central role in tumor progression and metastasis [34]. The findings of the present study suggest that the expression of ICAM-1 in DLD-1 cells may enhance their adhesiveness to HUVECs: 1) ICAM-1 was up-regulated by SDF-1 stimulation in DLD-1 cells; 2) functional blockade of ICAM-1 by siRNA and neutralizing antibody resulted in markedly decreased adherence of DLD-1 cells to HUVECs.

The expression of SDF-1 has been detected in several cancer cells, indicating that these pathways may be important in the metastasis of tumor cells [35,36]. SDF-1 is a constitutively expressed and inducible chemokine that modulates biological and physiological processes, including embryonic development and organ homeostasis [37]. Abnormal expression of SDF-1 has been detected in various solid tumor tissues such as ovarian cancer [38], breast cancer [39] and colorectal cancer [40]. SDF-1 has been shown to promote the growth, invasion and metastasis of CRC cells [41], implying that SDF-1 plays a role in the progression of CRC; however, the mechanisms of how SDF-1 stimulates metastasis of CRC cells are not totally understood. In this study, we evaluated the molecular mechanisms underlying the roles of SDF-1 in modulating ICAM-1 expression and cell adhesion to HUVECs in colon cancer cells. Our results have demonstrated that SDF-1-induced expression of ICAM-1 expression is mediated by the MAPKs, and downstream Sp1, C/EBP-β and NF-κB signaling pathways.

The promoter region of the ICAM-1 gene has different binding sites for various transcriptional factors [42]. Previous studies have shown that the transcription factors NF-κB, Sp1 and C/EBP-β can be activated through the MAPK pathways in colorectal cancer cells [20,43,44]. Regulation of gene expression through the use of combinations of different transcription factors has been observed [45,46]. It has been reported that NF-κB and Sp1 can be activated by MAPK family members, depending on the target gene in different cells [47]. Patel et al. reported that MAPKs mediate NF-κB and C/EBP-β activation, which leads to the transcription of specific genes [48]. To evaluate the signaling factors upstream of the activation of NF-κB, Sp1 and C/EBP-β that lead to ICAM-1 transcriptional activation in DLD-1 cells, we investigated the role of MAPKs using specific inhibitors. In addition, we performed TF ELISA assays to demonstrate that the regulation of ICAM-1 gene expression in DLD-1 cells was mediated by increased NF-κB p65, Sp1- and C/EBP-β-DNA binding activities. Based on our results, we propose a possible signal transduction pathway in DLD-1 cells in which SDF-1 induces ERK, JNK and p38 phosphorylation, which activates NF-κB, Sp1 and C/EBP-β to lead to their binding to respective sites in the ICAM-1 promoter, thus resulting in ICAM-1 transcriptional activation.

Several studies have indicated that CRC cells express CXCR4 and CXCR7, the receptors for SDF-1, and that their survival and migration to distant tissues are promoted by SDF-1 [41,49]. The binding of SDF-1 to CXCR4 may affect several signaling pathways, which can lead to multiple responses. Our previous study revealed that the expression of urokinase plasminogen activator in CRC DLD-1 cells by SDF-1 stimulation is mediated by CXCR4 [20]. In order to analyze the role of CXCR4 in ICAM-1 expression in human CRC cells, we evaluated the effect of CXCR4 function-blocking antibody on SDF-1–induced ICAM-1 expression. Our results suggest that when DLD-1 cells encounter an external stimulus of SDF-1, SDF-1 induces ICAM-1 expression via CXCR4 up-regulation. Interestingly, DLD-1 cells did not express surface CXCR4 [50], but they had abundant CXCR4 after SDF-1 stimulation, as determined by flow cytometry. Up-regulation of CXCR4 cells is functionally important, as SDF-1-stimulated DLD-1 cells in contrast to control cells respond to SDF-1 by inducing the ICAM-1 expression and adhering to HUVECs in response to SDF-1. Previously it had been reported that CXCR4 surface expression is hardly detectable on neutrophils, but expression became apparent gradually after incubation [51]. An alternative possibility described recently postulates that CXCR7 may serve as a co-receptor for CXCR4 and mediate SDF-1–induced intracellular signaling [52]. Several studies have indicated that CRC cells also express CXCR7, the receptors for SDF-1, and that their survival and migration to distant tissues is promoted by SDF-1 [53]. However, the mechanisms of CXCR4 expression in DLD-1 cells and the interaction between CXCR7 remains unknown, and further study is required to identify whether SDF-1 affects CXCR7 function and CXCR4 expression in CRC cells.

ICAM-1 expression modulates several cell functions and processes that regulate the progression of malignancies, including apoptosis, cell motility, invasion and angiogenesis. It has been shown that ICAM-1 may support mesothelial adhesion of CRC cells [11]. Interaction of ICAM-1 on vascular or lymphatic endothelial cells has been found to promote the adhesion of lung cancer and breast cancer cells [54,55]. In addition, ICAM-1 may be expressed by cancer cells themselves and contribute to their invasiveness [56].

## Conclusions

In summary, our data contribute new information about the mechanisms by which SDF-1 induces ICAM-1 expression and cell adhesion to the endothelium in colon cancer cells.

## Abbreviations

CRC: Colorectal cancer; CXCL12: CXC chemokine ligand 12; SDF-1: Stromal cell-derived factor; CXCR4: CXC receptor 4; ICAM-1: Intercellular adhesion molecule-1; MAPKs: Mitogen-activated protein kinases; HUVEC: Human umbilical vein endothelial cells; ChIP: Chromatin immunoprecipitation assay.

## Competing interests

The authors declare that they have no competing interests.

## Authors’ contributions

S-YT and C-NC designed research; S-YT, S-FC, M-HC, W-SH, Y-YH, and C-HS performed research; S-YT, S-FC and C-NC analyzed data; and H-CK and C-NC. wrote the paper. All authors read and approved the final manuscript.
