# Correction: CXC chemokine ligand 12/Stromal cell-derived factor-1 regulates cell adhesion in human colon cancer cells by induction of intercellular adhesion molecule-1

**DOI:** 10.1186/1423-0127-20-21

**Published:** 2013-03-26

**Authors:** Shui-Yi Tung, Shun-Fu Chang, Ming-Hui Chou, Wen-Shih Huang, Yung-Yu Hsieh, Chien-Heng Shen, Hsing-Chun Kuo, Cheng-Nan Chen

**Affiliations:** 1 Department of Hepato-Gastroenterology, Chang Gung Memorial Hospital, Chiayi, Taiwan; 2 Chang Gung University College of Medicine, Taoyuan, Taiwan; 3 Biophotonics & Molecular Imaging Research Center, National Yang Ming University, Taipei, Taiwan; 4 Department of Biochemical Science and Technology, National Chiayi University, Chiayi, Taiwan; 5 Division of Colon and Rectal Surgery, Department of Surgery, Chang Gung Memorial Hospital, Chiayi, Taiwan; 6 Department of Nursing, Chang Gung University of Science and Technology; Chronic Diseases and Health Promotion Research Center, CGUST, Chiayi, Taiwan

## Correction

After publication of this work [1], we noted that we inadvertently failed to add the affiliations of first author Shui-Yi Tung. The author has now been added.

## Competing interests

The authors declare that they have no competing interests.

## Authors’ contributions

S-YT and C-NC designed research; S-YT, S-FC, M-HC, W-SH, Y-YH, and C-HS performed research; S-YT, S-FC and C-NC analyzed data; and H-CK and C-NC wrote the paper. All authors read and approved the final manuscript.
